# PLCG2 modulates TREM2 expression and signaling in response to Alzheimer's disease pathology

**DOI:** 10.1002/alz70855_104521

**Published:** 2025-12-24

**Authors:** Evan J Messenger, Sydney Baar, Logan M Bedford, Andy P Tsai, Peter Bor‐Chian Lin, Guixiang Xu, Abigail Wallace, Gary E Landreth, Bruce T. Lamb, Stephanie J Bissel

**Affiliations:** ^1^ Indiana University School of Medicine, Indianapolis, IN, USA; ^2^ Wu Tsai Neurosciences Institute, Stanford University, Stanford, CA, USA; ^3^ Washington University in St. Louis, St Louis, MO, USA; ^4^ Indiana University, Stark Neurosciences Research Institute, Indianapolis, IN, USA; ^5^ Stark Neurosciences Research Institute, Indiana University School of Medicine, Indianapolis, IN, USA

## Abstract

**Background:**

Many of the genes associated with altered risk for Alzheimer's disease (AD) are predominantly expressed in microglia and affect innate immune responses. Among these genes is phospholipase C gamma 2 (PLCG2), a mediator of transmembrane signaling that acts downstream of immune receptors on microglia, including TREM2. Transcriptomic studies suggest a vital role for PLCG2 in the immune response, learning, and metabolism. Recent studies have identified genetic variants of PLCG2 which bidirectionally alter AD risk.

**Methods:**

To elucidate PLCG2‐dependent mechanisms relevant to these divergent effects, we explored the impact of PLCG2 ablation on amyloid pathology and microglial response in the amyloidogenic 5xFAD murine model of AD. In addition, to elucidate PLCG2's role in TREM2 signaling we also dissected functional and transcriptomic effects of TREM2 deficiency.

**Results:**

PLCG2 deficiency resulted in a reduction of TREM2 gene and protein expression, while TREM2 deficiency did not impact PLCG2 expression, suggesting an important role for PLCG2 in the induction of TREM2 in AD. Moreover, human cortical bulk RNA‐Seq shows that *PLCG2* and *TREM2* expression strongly correlate, regardless of clinical pathological scores, indicating that the relationship between PLCG2 and TREM2 expression might be biological and not depend on degree of disease or microgliosis. In 5xFAD mice, PLCG2 and TREM2 knockout similarly increased the LAMP1 area per plaque, potentially indicating an increase in plaque neurotoxicity. PLCG2 knockout microglia showed a significant reduction in phagolysosomal marker CD68 compared to TREM2 knockout, hinting at a potential difference in plaque phagocytosis and lysosomal processing. Transcriptomic analysis of PLCG2 and TREM2 knockout mice revealed many similarly enriched pathways involving the immune response. The overlapping DEGs are highly enriched for the immune response and both genotypes show similar reductions in many DAM marker genes. However, the PCA plot reveals that the PLCG2 and TREM2 knockouts are transcriptionally distinct, with pathways involving cell migration, adhesion, and proliferation being enriched in the DEGs between them.

**Conclusion:**

Overall, this study highlights the importance of PLCG2 in the innate immune response to amyloid pathology and TREM2 signaling and reveals key pathways which may clarify PLCG2's role in microglial functioning in AD.

